# Assessment of Serum 25-Hydroxyvitamin D and Its Association in Type 2 Diabetes Mellitus Elderly Patients with Kidney Disease: A Retrospective Cross Sectional Study

**DOI:** 10.3390/metabo13030357

**Published:** 2023-02-28

**Authors:** Moyad Shahwan, Nageeb Hassan, Noor Mazin, Ammar Jairoun, Sahab Al Khoja, Monzer Shahwan, Osama Najjar, Tariq Al-Qirim

**Affiliations:** 1 Department of Clinical Sciences, College of Pharmacy and Health Sciences, Ajman University, Ajman P.O. Box 346, United Arab Emirates; 2 Centre of Medical and Bio-Allied Health Sciences Research, Ajman University, Ajman P.O. Box 346, United Arab Emirates; 3 School of Pharmaceutical Sciences, University Sains Malaysia (USM), Pulau Pinang 11500, Malaysia; 4 Health and Safety Department, Dubai Municipality, Dubai P.O. Box 67, United Arab Emirates; 5 Diabetes Clinic, AL-Swity Center for Dermatology and Chronic Diseases, Ramallah 972, Palestine; 6 General Directorate of Allied Health Professions, Ministry of Health, Nablus 972, Palestine; 7 Faculty of Pharmacy, Al-Zaytoonah University of Jordan, P.O. Box 130, Amman 11733, Jordan

**Keywords:** vitamin D deficiency, kidney disease, type 2 diabetes mellitus, 25-hydroxyvitamin D

## Abstract

The overall aim of this study is to determine the prevalence of vitamin D deficiency and its association with diabetic nephropathy in elderly patients with type 2 diabetes mellitus. This study is a single center retrospective cross-sectional design conducted at private medical center. The study group included all patients (18 years or older) suffering from type 2 diabetes mellitus that attended the diabetic clinic from September 2019 to January 2021. The main outcome variable is a trough level of (<20 ng/mL) for 25OHD. The patients were categorized as having diabetic nephropathy based on estimated glomerular filtration rate (eGFR). Total glycated hemoglobin (HbA1c), creatinine serum, Alb: Cr ratio, total cholesterol (TC), triglyceride (TG), high-density lipoprotein (HDL-C), low-density lipoprotein (LDL-C), systolic blood pressure (SBP) and diastolic blood pressure (DBP) were compared between vitamin D deficiency groups. Univariate and multivariate logistic regression was used to investigate the association between vitamin D deficiency and other significant anthropometric and biochemical factors. A *p* value < 0.05 was chosen as the criterion to make decisions regarding statistical significance. Among the 453 diabetic patients included in study, 48.6% (*n* = 220) were male and 51.4% (*n* = 233) were female. The mean age ± S.D of the patients was 54.5 ± 10.6 years old. Out of 453 diabetic patients, 71.1% (95% CI: 66.9%–75.3%) had vitamin D deficiency (25OHD < 20 ng/mL). There was a statistically significant association between 25OHD level and diabetic nephropathy in elderly patients with type 2 diabetes mellitus. Diabetic patients with e-GFR < 60 mL/min more likely to have vitamin D deficiency (*p* < 0.001). Similarly, individuals with Alb: Cr ratio > 30 mg/g were more likely to have vitamin D deficiency (*p* < 0.001). Moreover, diabetic patients with serum creatinine > 1.8 mg/dL were more likely to have vitamin D deficiency (*p* < 0.001). The study revealed a high prevalence of vitamin D deficiency in elderly patients with type 2 diabetes mellitus. A significant association was reported between 25-hydroxyvitamin D, e-GFR and Alb: Cr ratio.

## 1. Introduction

Vitamin D deficiency (VDD) is a growing global public health problem. Various investigators have examined different cutoffs for vitamin D status, but the majority define VDD as serum 25(OH)D levels < 20 ng/mL and serum 25(OH)D levels between 20 and 20 ng/mL. We defined a deficit as 20 ng/mL. Around 1 billion people are affected worldwide, which is 15% of the world’s population. Vitamin D plays a key role in many important homeostatic processes, including bone metabolism, cell proliferation, neuromuscular and immune function, and inflammation. As a result, low serum vitamin D levels predispose people to a variety of health complications, including multiple sclerosis, autoimmune diseases, infectious diseases, respiratory disease, cardiometabolic disease, and cancer [1].

However, epidemiological findings regarding the association of VDD with numerous clinical manifestations, particularly type 2 diabetes mellitus (T2DM), remain conflicting. Several longitudinal observational studies and their systematic reviews and meta-analyses support the assumption that VDD may increase the risk of type 2 diabetes and associated complications [2,3].

Diabetes is considered one of the growing epidemics worldwide; its prevalence has increased over and over, and it is expected to become the main cause of morbidity and mortality [4]. This high rate of morbidity and mortality is due to macrovascular and microvascular complications [5]. Diabetic kidney disease (DKD) is the most common microvascular complications in type 2 diabetic patients [6]. Approximately 40% of diabetic patients are at end stage renal disease [7]. Most of these patients have vitamin D deficiency [8].

DKD is characterized by hypertrophy of the glomerular and tubular epithelium, basement membrane thickening, and extracellular matrix deposition, ultimately leading to glomerulosclerosis and tubulointerstitial fibrosis. The major clinical indications of DKD are progressive proteinuria and declining renal function. Risk factors for DKD include genetic polymorphisms, prolonged hyperglycemia, obesity, hypertension, and dyslipidemia. Pathophysiological changes in DKD are likely due to metabolic and hemodynamic abnormalities. However, the exact underlying mechanisms are complex and may involve multiple pathways. Studies have shown that when the intrarenal renin-angiotensin system ‘RAS’ is activated, it plays an important role in causing progressive renal damage in DKD [9].

Taking medications and implementing lifestyle changes such as weight loss, dietary modifications, smoking cessation and exercise will improve patients’ glycemic control, and hence will minimize diabetes complications [10]. In addition, supplementation with vitamin D will improve glycemic control in patients with diabetes type 2 [11].

Vitamin D receptors exist in the B-cell of the pancreas [12]; vitamin D is then hydroxylated in the liver into 25-hydroxyvitamin D (25(OH)D), and in the kidney into 1,25-dihydroxyvitamin D (1,25(OH)2D), and these metabolites will bind with the vitamin D receptors in the pancreas [13,14].

Vitamin D plays an important role in diabetes and its complications, it has been shown in some studies that vitamin D supplementation could decrease the occurrence of diabetes [15]. A low level of vitamin D l is a risk factor for glucose metabolism distraction and diabetes type 2 [16]. Mainly, vitamin D deficiency is due to low exposure to sunlight, skin color, and a low nutritional supply of food containing vitamin D [17]. An experimental study shows that diminished glucose-facilitated insulin secretion in the situation of vitamin D deficiency could be treated and minimized by taking vitamin D supplementation [18].

There are different assumptions regarding the role of vitamin D in diabetes. One study showed that vitamin D protects β-cells from death [19]; another one showed vitamin D may affect the growth and differentiation of β-cells [20]. The fact is that vitamin D is a main regulator of the metabolism of calcium, hence it is important to supply an adequate concentration of calcium for bone mineralization. Therefore, vitamin D is important for the metabolism of glucose, since calcium is critical for insulin secretion and synthesis [21].

Other study shows the effect of CKD on vitamin D levels. CKD patients are at increased risk of vitamin D deficiency for several reasons. The main reasons are as follows: loss of vitamin D binding protein (DBP), the major carrier protein for 25(OH)D, due to proteinuria in the nephrotic region associated with type 2 diabetes and certain types of glomerulonephritis. Patients should strictly limit foods containing vitamin D to avoid excessive phosphorus intake. They should have less sun exposure and, most importantly, significantly reduced expression of the endocytic receptor megalin in their renal proximal tubules. Renal megalin depletion occurs in the very early stages of CKD, and this condition is associated with decreased reabsorption of glomerular-filtered albumin and other low-molecular-weight proteins [22].

Diminishing vitamin D metabolism may be a new therapeutic goal to minimize the progression and development of DKD [23]. A study has shown that 25(OH) D concentration is lower in patients with diabetes and kidney disease compared to those patients without these two conditions [24]. Kidney disease affects vitamin D metabolism, hence monitoring of the vitamin D concentration in diabetic patients with nephropathy is important [25]. There is an inverse relation between concentration of vitamin D and albuminuria [26]. Several studies have found an opposite relationship between serum 25(OH) D concentration and the glycated haemoglobin (HbA1c) concentration in type 2 diabetic patients; this shows the need for the regular screening of vitamin D level among diabetic patients, to treat the depletion from the beginning in order to achieve better glycemic control and prevent development of complications from diabetes type 2 [27]. A cross-sectional study found that type 2 diabetic patients with kidney disease and vitamin D deficiency are have a significantly higher risk of developing cardiovascular disease compared with those with normal vitamin D levels [28]. Another study has the same result; it showed that low levels of vitamin D will increase the risk of cardiovascular disease as well as all-cause mortality and cardiovascular disease [29,30].

There are numerous studies showing the relationship between vitamin D and diabetes types; only a few have studied the relationship between these two and kidney diseases. Hence, in this study, we will focus on the relationship between vitamin D and kidney disease in type 2 diabetic patients.

Our aim in this study is to investigate the relationship between serum 25OHD and kidney disease in type 2 diabetic patients

## 2. Materials and Methods

### 2.1. Subjects, Materials, and Methods

This study is a retrospective cross-sectional design conducted at private medical center. The study group included all patients (18 years or older) suffering from type 2 diabetes mellitus attended the diabetic clinic from September 2019 to January 2021.

The participants were outpatients, consecutively recruited by specialist diabetes physician at regular follow up visits. It comprised a systematic sample of 453 type 2 diabetic patients, of which 220 and 233 were males and females, respectively. Patients’ confidentiality was respected; all patients were reviewed anonymously, and data was gathered using their identification numbers and coding. Furthermore, the data processing and data entry were handled only by the principal investigator, so there were no complications of harm to the patients.

Relevant sociodemographic and clinical and laboratory data were obtained from the medical records of the patients, including age, gender, height, weight, body mass index (BMI), total glycated hemoglobin (HbA1c), fasting blood glucose (FBS), medications history, c-reactive protein (CRP), creatinine, estimated glomerular filtration rate (eGFR), erythrocyte sedimentation rate (ESR), etc.

### 2.2. Data Collection Tool

The collection sheet used as a tool in this study included the following variables: age, gender, marital status, weight, height, educational level, smoking status, physical activity, BMI and blood pressure

Laboratory tests include total glycated haemoglobin (Hba1c), creatinine, glomerular filtration rate (GFR), ESR, CRP, blood urea nitrogen (BUN), creatinine urine, albumin/creatinine ratio, ALT, AST, total cholesterol, triglyceride, high density lipoprotein, low density lipoprotein, vitamin D, vitamin B12, calcium, albumin, potassium, and sodium.

Medication history was collected, including past and current medication in addition to the past and present co-morbidities for all the study participants.

### 2.3. Sample Size Calculation

The assumed prevalence of vitamin D deficiency among type 2 diabetes patients was 66%. An alpha level of 5% was selected for this study, meaning a 95% confidence interval (CI). With the precision (D) of 5%, 10% was taken to be the maximum width of the 95% CI. Thus, 435 patients were considered to be a sufficient sample size for the final study, assuming missing patients data at baseline of 24%.

### 2.4. Inclusion Criteria

Male and female patients with type 2 diabetes and with chronic renal disease, age 18 or above were included in the article, irrespective of their vitamin D levels

Laboratory tests for inflammatory markers such as CRP and ESR, as well as renal function tests such as glomerular filtration rate, albumin, creatinine, and BUN were performed on patients.

### 2.5. Exclusion Criteria

Male and female patients with type 1 diabetes, patients without renal disease, patients aged 17 or under, pregnant females and children were excluded. In addition, the study excluded patients with renal disease unrelated to diabetes.

### 2.6. Statistical Analysis

The data were analyzed using the SPSS version 26. Frequencies and percentages were used to summarize the qualitative variables. Graphical representations were provided for all relevant variables. Evaluation of the distribution and normality of the data was carried out using a Shapiro–Wilk test (with *p* > 0.05 indicating a normally distributed continuous variable) or by visual inspection of a normal Q-Q Plot. Chi-square and Fisher’s exact tests were used to compare the difference between categorical variables. Univariate and multivariate logistic regression was used to investigate the association between the vitamin D deficiency and other significant anthropometric and biochemical factors. A backward and forward stepwise procedure was applied to the multivariate logistic regression model. A *p* value < 0.05 was chosen as the criteria to make decisions regarding statistical significance.

### 2.7. Ethical Approval

This study was approved by the Health and Ethics Review Committee of the participating healthcare center. All respondents gave their informed consent in accordance with the Declaration of Helsinki.

## 3. Results

### 3.1. Anthropometric and Biochemical Characteristics of the Subjects (n = 453)

Table 1 presents the anthropometric and biochemical characteristics of the study participants. Among the 453 diabetic patients included in study, 48.6% (*n* = 220) were male and 51.4% (*n* = 233) were female. The mean age ± S.D of the patients was 54.5 ± 10.6 years old. The mean ± S.D of HbA1c, creatinine, Alb: Cr ratio, e-GFR, TC, TG, HDL-C, LDL-C, SBP and DBP were 7.8 ± 1.3 (%), 0.84 ± 0.53 (mg/dL), 130.1 ± 14.61 (mg/g), 255.6 ± 21.3 (mg/dL), 180.1 ± 25.3 (mg/dL), 184.3 ± 25.4 (mg/dL), 41.4 ± 11.4 (mg/dL), 99.8 ± 41.5 (mg/dL), 129.4 ± 14.3 (mmHg), 80.8 ± 8 (mmHg), respectively.

### 3.2. Association between Vitamin D Deficiency and Renal Biochemical Markers (n = 453)

Out of 453 diabetic patients, 71.1% (95% CI: 66.9%–75.3%) had vitamin D deficiency (25OHD < 20 ng/mL). Prevalence of different types of diabetic nephropathy stratified by patients’ vitamin D levels are shown in Table 2. Patients were divided into two groups, as per 25-hydroxyvitamin D; the first group consisted of patients with 25OHD < 20 ng/mL, and the second group consisted of patients with 25OHD ≥ 20 ng/mL. There was a statistically significant association between 25OHD level and diabetic nephropathy in patients with type 2 diabetes mellitus. Diabetic patients with e-GFR < 60 mL/min more likely to have vitamin D deficiency (*p* < 0.001). Similarly, individuals with an Alb: Cr ratio >30 mg/g were more likely to have vitamin D deficiency (*p* < 0.001). Moreover, diabetic patients with serum creatinine > 1.8 mg/dL were more likely to have vitamin D deficiency (*p* < 0.001).

### 3.3. Evaluation of Risk Factors Associated with Vitamin D Deficiency in Type 2 Diabetes Mellitus

Table 3 displays the results of logistic regression analysis of the anthropometric parameters and biochemical markers that are associated with the vitamin D deficiency.

From the univariate analysis, gender (OR = 1.23, CI = 1.14–3.75, *p*-value = 0.031), age (OR = 1.45, CI = 1.11–2.03, *p*-value = 0.004), HbA1c (OR = 1.78, CI = 1.13–1.98, *p*-value = 0.001), serum creatinine (OR = 1.68, CI = 1.19–4.52, *p*-value = 0.017), e-GFR (OR = 0.26, CI = 0.154–0.44, *p*-value < 0.001), Alb: Cr ratio (OR = 0.96, CI = 0.93–0.97, *p*-value < 0.001), total cholesterol (OR = 1.49, CI = 1.01–1.77, *p*-value = 0.009), triglycerides (OR = 1.61, CI = 1.11–1.99, *p*-value = 0.042), LDL cholesterol (OR = 1.24, CI = 1.09–1.74, *p*-value = 0.004) and diastolic blood pressure (OR = 11.120, CI = 1.069–1.173, *p*-value = 0.027) are strong determents of vitamin D deficiency.

To select the set of factors that jointly influence vitamin D deficiency, we used the backward and forward stepwise procedure applied to the multivariate logistic regression model. The results of this procedure showed that HbA1c, e-GFR and mean Alb: Cr ratio are jointly highly associated with vitamin D deficiency. If the HbA1c increases by 1%, then the odds of having 25OHD < 20 ng/mL increase by 71%. If the e-GFR increases by 1 mL/min, then the odds of having 25OHD < 20 ng/mL decrease by 70%. If the Alb: Cr Ratio increases by 1 mg/g, then the odds of having 25OHD < 20 ng/mL decrease by 6% (Table 3).

## 4. Discussion

The current study specifically targeted T2DM with serum 25(OH)D levels and CKD. The main finding of this study was that lower serum 25(OH)D levels were significantly associated with an increased risk of CKD progression in type 2 diabetic patients. According to recent studies, there is a high prevalence of 25(OH)D deficiency around the world due to aging, diet, low physical activity and sun exposure, among other factors [31,32,33].

The present retrospective article aims to study the relation between the 25OHD and diabetic nephropathy in elder people with type 2 diabetes mellitus. Patients with kidney failure demonstrated a considerable association with the measured levels of 25OHD levels, as the patients with low eGFR had higher chances of developing vitamin D deficiency. The same results matched the results in Sung Gil Kim’s article, as it was found that the likelihood of having a vitamin D deficiency along with decreased eGFR is high [34]. Moreover, Jong Park had stated in his study that the levels of the estimated GFR decrease significantly when the levels of 25OH D levels are increased [35]. This relationship is generally shown by the fact that a glomerular filtration rate decrease results in a decreased amount of 1-α-hydroxylase substrate delivery. The later action limits the capability of the kidney to form 1,25-dihydroxyvitamin D [36]. Experimental results demonstrate that vitamin D is an effective inhibitor of the of the renin-angiotensin system (RAS) and nuclear factor (NF)-kB pathway, and in human trials, low levels of vitamin D have been autonomously linked to higher plasma renin and angiotensin 2 concentrations. These pathways play a significant role in the disease processes of kidney disease through mediating the immune, inflammatory, and proliferative effects that result in progressive renal damage. A 25(OH)D deficiency may accelerate the progression of albuminuria, which is by itself a recognized indicator of CKD progression and adverse cardiovascular outcomes, as well as the decline in renal function [37,38,39].

Age-related decreases in the functioning of kidneys have been shown to be associated with a decrease in the synthesis, metabolism, and transportation of 1,25 dihydroxyvitamin D. Patients with ESRD have a higher prevalence of vitamin D deficiency [9,40,41].

The current study revealed a high prevalence of vitamin D deficiency in elderly patients with type 2 diabetes mellitus. A significant association was reported between 25(OH)D, e-GFR and the Alb: Cr ratio.

A study by Shaofeng et al. analyzed the relationship between albuminuria 25(OH)D, and concluded in their results that a low level of vitamin D is a major indicator of incident nephropathy in type 2 diabetic patients, and the prevalence of vitamin D deficiency in patients with albuminuria was greater than the patients without it [42].

Moreover, the odds ratio between the vitamin D levels and the Alb: Cr ratio were inversely related. Individuals with high levels of albumin excretion in urine had reduced recorded vitamin D levels. It has been found that after a 6 months’ therapy with vitamin D to diabetic nephropathy patients, the urine albumin, renin and serum creatinine levels were reduced, leading to a noticeable improvement of GFR for these patients. There are signs to researched further that high vitamin D treatments can be reno-protective [43].

Alborzi et al.’s double-blind randomized clinical trial revealed that a half amount of albuminuria was reduced by the paricalcitol treatment for a solid month. At the end of the time for which the trial took place, the paricalcitol groups treated with 1 μg and 2 μg had 0.52 and 0.54 levels of albumin compared to the baseline levels at the beginning of the study, whereas the placebo groups showed an increase by 1.35 times compared to the same baseline. Nevertheless, the small sample size (consisting of eight sample sizes in each group) is the major drawback of this study [44].

Another trial, which included the administration of calcitriol using 0.5 μg two times a week for a length of 12 consecutive years, in 10 patients resulted in a major reduction of the urine albumin to creatinine ratio, proved by IgA nephropathy. However, the small sample size and the absence of placebo are the drawbacks of this trial [45].

Mao et al. showed in their prospective study that the additional supply of calcitriol resulted in reduction of the protein in the diabetic patients. This study was performed on patients with vitamin D deficiency and insufficiency [46].

On the other hand, Alexander Teumer et al. have found that circulating levels of vitamin D negatively relate to eGFR levels. However, the small sample size and exclusion of meta-analysis studies were the major drawbacks of this study. Further studies are needed to establish the underlying relation [47].

As expected, our article showed that diabetic patients are more likely to have vitamin D deficiency when their serum creatinine levels > 1.8 mg/dL.

Regarding our study, certain factors were found to be associated with vitamin D deficiency. HbA1c, e-GFR and mean Alb: Cr ratio are together linked to vitamin D deficiency. On the other hand, in the study by Tricia L Larose [48], the resulting factors were found to be winter season, BMI and dietary intake, in order. The winter season was the most related factor in many other studies too [49,50].

However, another report showed that vitamin D deficiency is prevalent in countries with lots of sunshine such as Saudi Arabia. Younger women were associated with vitamin D hypovitaminosis to a larger degree than older women, which was explained by the increased quantity of supplemental vitamin D intake by the older women [50].

One of the limitations that may restrict the generalizability of the findings is that the research was performed in only one geographic area, so no long-term conclusions can be drawn, and the results may not extend to other regions. Results may differ for other countries due to the presence of factors that could affect vitamin D levels, hence additional investigations should be carried out for other countries. Secondly, this study was a cross-sectional study design. Thirdly, levels of serum insulin were not measured for the candidates. Fourth, serum glucose/insulin levels were not determined for type 2 patients, but these may affect the result of vitamin D levels in another patients.

## 5. Conclusions

This study revealed a high prevalence of vitamin D deficiency in elderly patients with type 2 diabetes mellitus. A significant association was reported between 25-hydroxyvitamin D, e-GFR and Alb: Cr ratio. Therefore, regular checkups for vitamin D levels in elderly patients are necessary to maintain glycemic control and prevent development of complications, especially CKD. Further studies are needed to clarify the relationship between vitamin D and renal biochemical markers (glomerular filtration rate, albumin, creatinine, and BUN), and to illuminate the scientific support for prevention and treatment of CKD. In future works, to further investigate the utility of screening 25-hydroxyvitamin D, it would be useful to know if regular check-ups of 25-hydroxyvitamin D and long-term maintenance of an optimal 25 hydroxyvitamin D level could be helpful to achieve better glycemic control and prevent development of diabetes type 2 complications, especially kidney diseases.

## Figures and Tables

**Table 1:** Anthropometric and biochemical profile parameters results for patients with T2DM.

Parameters	All Patients (*n* = 453)		
	Mean	±SD	Median
Age(years)	54.5	10.6	56
HbA1c (%)	7.8	1.3	7.6
Creatinine serum mg/dL	0.84	0.53	0.78
Alb: Cr Ratio mg/g	130.1	14.61	127.54
e-GFR (mg/dL)	255.6	21.3	220.4
TC (mg/dL)	180.1	25.3	182
TG (mg/dL)	184.3	25.4	167
HDL-C (mg/dL)	41.4	11.4	40
LDL-C (mg/dL)	99.8	41.5	97
SBP (mmHg)	129.4	14.3	120
DBP (mmHg)	80.8	8	80
Vitamin D (20 ng/mL)	21.2	22.3	21.9
Gender	Male	220	48.6%
	Female	233	51.4%

Abbreviations: HbA1c, hemoglobin A1c; HDL-C, high-density lipoprotein cholesterol; LDL-C, low-density lipoprotein cholesterol; TC, total cholesterol; TG, triglyceride, e-GFR; glomerular filtration rate, Alb: Cr; albumin to creatinine ratio, SBP, systolic blood pressure; DBP, diastolic blood pressure.

**Table 2:** Renal biochemical markers according to vitamin D deficiency.

Renal Biochemical		Vitamin D Deficiency Status		*p*-Values
	Groups	25OHD < 20 ng/mL	25OHD ≥ 20 ng/mL	
e-GFR (mL/min)	<60 mL/min	76 (37.6%)	126 (62.4%)	<0.001
	>60 mL/min	246 (66.1%)	5 (6.2%)	
Alb: Cr ratio	>30 mg/g	200 (77.2%)	59 (22.8%)	0.001
	<30 mg/g	122 (62.9%)	72 (37.1%)	
Creatinine mg/dL	>1.8 mg/dL	31 (96.9%)	1 (3.1%)	0.001
	<1.8 mg/dL	291 (69.1%)	130 (30.9%)	

Abbreviation: 25OHD, 25-hydroxyvitamin D; e-GFR; glomerular filtration rate, Alb: Cr; albumin to creatinine ratio, *p* values less than 0.05 were considered statistically significant. Notes: *p*-values were obtained from Chi-square and Fisher’s exact tests.

**Table 3:** Regression analysis of factors associated with vitamin D deficiency among patients with type 2 diabetes.

Factors	Vitamin D = 25OHD < 20 ng/mL							
	Univariate Analysis				Multivariate Analysis			
	OR	95% CI		*p*-Value	OR	95% CI		*p*-Value
Sex (female vs. male)	1.23	1.14	3.75	0.031	---	---	---	---
Age (years)	1.45	1.11	2.03	0.004	---	---	---	---
HbA1c (%)	1.78	1.13	1.98	0.001	1.71	1.04	2.89	0.0016
Creatinine serum mg/dL	1.68	1.19	4.52	0.017	---	---	---	---
e-GFR (mL/min)	0.26	0.154	0.44	<0.001	0.301	0.157	0.575	<0.001
Alb: Cr Ratio mg/g	0.96	0.93	0.97	<0.001	0.94	0.92	0.96	<0.001
TC (mg/dL)	1.49	1.01	1.77	0.009	---	---	---	---
TG (mg/dL)	1.61	1.11	1.99	0.042	---	---	---	---
HDL-C (mg/dL)	0.99	0.980	1.015	0.77	---	---	---	---
LDL-C (mg/dL)	1.24	1.09	1.74	0.004	---	---	---	---
SBP (mmHg)	1.04	0.96	1.12	0.309	---	---	---	---
DBP (mmHg)	1.120	1.069	1.173	0.027	---	---	---	---

Notes: *p* values less than 0.05 were considered statistically significant, “---” not included in the multivariate logistic regression model Abbreviations: OR, odds ratio; CI, confidence interval; HbA1c, hemoglobin A1c; HDL-C, high-density lipoprotein cholesterol; LDL-C, low-density lipoprotein cholesterol; TC, total cholesterol; TG, triglyceride, e-GFR; glomerular filtration rate, Alb: Cr; albumin to creatinine ratio, SBP, systolic blood pressure; DBP, diastolic blood pressure.

## Data Availability

The data presented in this study are available on request from the Corresponding author. Data is not publicly available due to privacy.
